# Electromyographic Diagnostic Ranges Defining Temporomandibular Disorders and Healthy Individuals’ Results in Functional Clenching Index

**DOI:** 10.3390/jcm14010014

**Published:** 2024-12-24

**Authors:** Grzegorz Zieliński, Michał Ginszt

**Affiliations:** 1 Department of Sports Medicine, Medical University of Lublin, 20-093 Lublin, Poland; 2 Department of Rehabilitation and Physiotherapy, Medical University of Lublin, 20-093 Lublin, Poland; michal.ginszt@umlub.pl

**Keywords:** electromyography, sEMG, masticatory muscles, functional indices, temporal muscle, masseter muscle, TMD, diagnostic, ranges

## Abstract

**Background**: Temporomandibular disorders (TMDs) represent a significant public health issue, among which masticatory muscle pain is the most common. Current publications increasingly indicate surface electromyography (sEMG) as an effective diagnostic tool for muscle dysfunctions that may be employed in TMDs recognition. The objective of this study was to establish reference ranges for TMDs patients with masticatory muscle pain and healthy individuals in the electromyographic Functional Clenching Index (FCI) for the temporalis muscles (TAs) and masseter muscles (MMs). This research aimed to provide an additional diagnostic tool for TMDs patients. **Methods**: A total of 48 individuals (*n* = 30 women and *n* = 18 men) with the muscular painful form of TMDs were recruited alongside a numerically and gender-matched control group—healthy, pain-free controls. The Functional Clenching Indexwas calculated for both groups. **Results**: Data analysis revealed statistically significant differences with a very large effect size. Healthy individuals had higher FCI scores compared to those with TMDs. The healthy group exhibited higher threshold values compared to the TMDs group. **Conclusions**: For healthy individuals, the FCI ranges for TAs were between 58 and 145, while for MMs, between 72 and 210. Lower values may indicate muscle activation disorders and occur in patients with the painful, muscular form of TMDs. This is the first study to define reference ranges for electromyographic indices; therefore, caution is recommended, and the replication of this study on a larger and more culturally diverse sample is advised.

## 1. Introduction

Temporomandibular disorders (TMDs) constitute a significant public health issue, affecting 34% of the global population. The term TMDs describes pain and dysfunction in the chewing muscles, temporomandibular joint, and surrounding tissues [1]. The etiological factors associated with TMDs can be divided into four groups: behavioral factors, social factors, emotional factors, and cognitive factors [2].

The first group includes, among others, bruxism (sleep and awake) [3,4]. Social factors influence the perception and the effect of learned responses to pain [2]. Emotional factors are related to psychological states, such as anxiety and depression [5,6]. Cognitive factors encompass negative thoughts and attitudes [2].

Currently, it is accepted that temporomandibular joint dysfunction typically involves an accumulation of factors leading to dysfunction. However, there are cases where a single factor can cause the disorder [7].

Genetic factors are also associated with TMDs [8,9]. Hormonal factors, on the other hand, are linked to the worsening of symptoms and increased pain response [10,11].

The number of factors influencing the temporomandibular joint, as well as those contributing to the development and exacerbation of TMDs, may explain why, under similar circumstances, one person’s TMJ may exhibit more severe clinical symptoms, while another’s does not [12].

Due to its multifactorial etiology and sometimes controversial etiologies [12], various diagnostic methods are employed in TMDs studies [1,2], including standardized approaches like the Diagnostic Criteria for Temporomandibular Disorders (RDC/TMD) [13] and the Diagnostic Criteria for TMD (DC/TMD) [14]. Rapid screening tools such as the pain screening questionnaire have also been developed [15]. The most common TMD symptom is myalgia within masticatory muscles, with a frequency of 30% [16]. Hence, additional objective diagnostic methods are needed to examine changes in muscle activity in patients with TMDs.

It is worth noting that TMDs are also associated with economic challenges. The treatment of TMDs involves significant expenses for patients as well as for national budgets [17]. The treatment of TMDs creates an annual economic burden of approximately USD 4 billion worldwide [18].

Surface electromyography (sEMG) was first introduced in dentistry in 1949, and the number of such studies has steadily increased [19]. Surface electromyography is a technique based on capturing, recording, and analyzing myoelectric signals generated by physiological changes in the membrane state of muscle fibers [20].

In electromyographic studies, data based on the amplitude and frequency of the signal are used to analyze the sEMG signal [21,22]. The raw amplitude data are normalized using the root mean square (RMS) algorithm for electromyography data analysis. RMS calculates the average power of the myoelectric signal, thereby eliminating the impact of short-term fluctuations [23]. This provides a more accurate picture of muscular activity, allowing for a better evaluation of muscle function during chewing, biting, and other activities related to the stomatognathic system. For this reason, it is frequently used in analyses of the bioelectric activity of masticatory muscles [22,24,25,26].

Various electromyographic indices based on RMS sEMG amplitude calculations are used within masticatory muscles for comparing results between patients and healthy individuals, including the Activity Index (AcI), Asymmetry Index (AsI) [27], and Percentage Overlapping Coefficient (POC) [28]. In 2021, the Functional Indices were developed, including the Functional Clenching Index (FCI) and Functional Opening Index (FOI) [26]. The FCI relies on resting and functional muscle activity, which may increase its effectiveness in analyses involving gender and other anthropometric variables [26].

Based on the above information, a study was conducted to determine the reference ranges of the FCI for TMDs patients and healthy individuals in the temporalis (TAs) and masseter muscles (MMs). Specifying these data may introduce and provide an additional diagnostic tool for TMDs patients.

## 2. Materials and Methods

This study was conducted with the approval of the local bioethics committee (KE-0254/25/01/2024). This research was divided into phases.

The first phase of this study involved informing participants about the research procedure, highlighting the specific aspects related to sEMG. Participants were informed that this study is safe and painless, and that they could withdraw at any point. Subsequently, in this phase, they were asked to complete documentation related to their consent to participate in this study.

The second phase of this study involved analyzing the presence of TMDs, as well as inclusion and exclusion criteria for this study. Detailed information regarding these criteria is provided below. Participants who reported pain symptoms were asked to complete the VAS scale.

The third phase of this study consisted of an electromyographic examination.

The fourth phase involved statistical analysis, with participants divided into two groups: healthy and those with TMDs.

Initially, 48 participants (*n* = 30 women and *n* = 18 men) with a muscular form of temporomandibular disorders (TMDs) were recruited based on the RDC/TMD diagnostic criteria [29]. The average level of pain measured using the VAS scale was determined to be 6 points, with a standard deviation of ±2 VAS [30]. A control group was selected to match the experimental group in terms of size and sex distribution. Participants in the control group were confirmed to be free of TMDs, according to RDC/TMD standards. Both groups were comparable in age, with the experimental group averaging 30 ± 8 years and the control group, 30 ± 10 years. Statistical analysis showed no significant age differences between groups (Z = −1.18, *p* = 0.24).

The main inclusion criteria for this study were as follows: age between 20 and 40 years; inclusion of both women and men who provided consent, with four support zones in the dental arch; and no history of surgery or injuries within the last six months.

Both groups were subjected to the following criteria: exclusion of individuals with malocclusions (Angle’s Class II or III); exclusion of those undergoing orthodontic treatment; exclusion of individuals with injuries or surgeries involving the head or neck within the past six months; exclusion of individuals with metal implants, regardless of type or location; exclusion of participants whose hair prevented electromyographic measurements; and exclusion of pregnant individuals.

### 2.1. Study Design Description

The objective of this study was to establish reference ranges for the FCI, derived from electromyographic measurements of the temporalis muscles and masseter muscles. The aim was to enhance diagnostic tools for patients with temporomandibular disorders characterized by masticatory muscle pain.

To ensure a structured approach, this study followed the following steps:

1. Participants were recruited and informed about the study procedure, including consent collection.
2. Participants were analyzed based on inclusion and exclusion criteria as well as for the presence of TMDs.
3. sEMG measurements of the TAs and MMs were performed.
4. Advanced statistical analyses were conducted to interpret the data and establish reference ranges.

This study included two groups:

- Healthy Group: Participants without TMDs or masticatory pain.
- TMD Group: Participants with TMDs characterized by muscle pain.

After screening for inclusion and exclusion criteria, sEMG examinations were conducted. Functional Clenching Index values were calculated for both the temporalis and masseter muscles. These indices are based on the ratio of muscle activity during clenching to resting muscle activity [26].

The obtained FCI values for both muscle groups were compared between the two groups. Advanced statistical analyses were used to determine ranges that could effectively differentiate healthy individuals from those with TMDs, contributing to improved diagnostic accuracy.

### 2.2. Surface Electromyography Procedure

**Figure 1:**
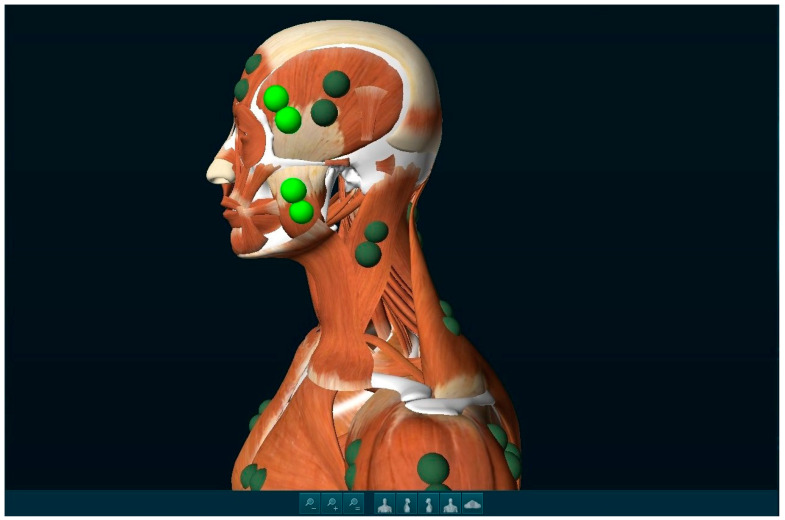
Electrode arrangement during surface electromyography. The green circles indicate the placement of the electrodes, and the graphic is sourced from dedicated electromyographic signal analysis software, Noraxon MR3 3.18.08 software (Noraxon USA, Inc., Scottsdale, AZ, USA).

Before the measurements, the skin was cleaned with 90% alcohol. The tests were conducted between 8:00 and 12:00 AM [19]. Electrodes were placed according to the SENIAM protocol (surface EMG for non-invasive assessment of muscles) [31]. The electrodes used were Ag/AgCl with a diameter of 30 mm and a conductive surface of 16 mm. The electrodes were placed symmetrically on both sides by the same individual, ensuring that the edges of the electrodes were in contact and that there was no hair beneath the conductive surface. The distance between the centers of adjacent electrodes was 30 mm. In accordance with the referenced protocol, the electrodes were positioned on the superficial part of the masseter muscle and the anterior part of the temporalis muscle. The presentation of electrode placement is shown in Figure 1. The placement of the electrodes was preceded by a palpatory examination conducted by a physiotherapist and an sEMG specialist (G.Z.). It was decided to adopt an approach in which an experienced physiotherapist additionally performed the palpation of the examined muscles to reduce the risk of so-called ‘cross talk’.

Cross-talk, in the context of sEMG studies, refers to interference in the electromyographic signal resulting from the recording of electrical activity from adjacent muscles instead of (or in addition to) the target muscle.

Typically, cross-talk constitutes 10–15% of the sEMG signal [20]. It occurs because surface electrodes capture electrical signals not only from the target muscle but also from other nearby muscles, especially if these muscles are active simultaneously.

While the risk of cross-talk was negligible in the context of the temporalis muscle, for the masseter muscle, it required the precise identification of the superficial muscle belly to minimize this risk.

Participants sat upright with their legs supported, torso aligned straight, and the head and neck in a straight position supported by a headrest [19]. The procedure involved recording resting activity for 10 s and functional activity, including clenching teeth (as hard as possible, 3 repetitions of 3 s, with 2 s intervals). Electromyographic data were recorded using the Noraxon Ultium DTS 8-K MR 3 myo Muscle Master Edition system (Noraxon USA, Inc., Scottsdale, AZ, USA). Settings included the following: sampling rate: 2000 Hz (motion: 200 Hz); high-pass filter cutoff: 10 Hz; low-pass filter cutoff: 500 Hz; input impedance: >100 MΩ; common mode rejection ratio: >100 dB; measurement accuracy: ±1% of the full scale [19]. Signal analysis was conducted using MR3 3.18.08 software, which involved raw signal cleaning and smoothing.

Next, the Root Mean Square, a standard algorithm for analyzing masticatory muscles in electromyographic studies [22,24,25], was calculated. The *RMS* is calculated using a program (Noraxon MR3 3.18.08 software (Noraxon USA, Inc., Scottsdale, AZ, USA) that is based on the following formula [23,32,33]:

$$RMS=\sqrt{\frac{1}{N}\sum_{i=1}^{N}x_{i}^{2}}$$

In the formula, *x_i_* represents the value of the signal at the *i*-th sample, while *N* is the number of samples in the analyzed period.

Electromyographic results were processed to the FCI using the following formula [26]:

$$Functional\;Clenching\;Index\;=\;\frac{clench}{rest}$$

The following mathematical formulas were adopted for the analysis:

Functional Clenching Index for TA right-sided (FCI_TA-R_) = CL_TA-R_/REST_TA-R_

Functional Clenching Index for TA left-sided (FCI_TA-L_) = CL_TA-L_/REST_TA-L_

Functional Clenching Index for MM right-sided (FCI_MM-R_) = CL_MM-R_/REST_MM-R_

Functional Clenching Index for MM left-sided (FCI_MM-L_) = CL_MM-L_/REST_MM-L_

### 2.3. Statistical Analysis

The minimum group size was determined based on previous studies [24]. Calculations were performed using G*Power software 3.1.9.7 (Heinrich-Heine-Universität Düsseldorf, Düsseldorf, Germany) [34], with the following assumptions: α = 0.05, power = 0.90, and effect size = 0.50 [35,36] for the *t*-test [24]. The required minimum sample size was 44 participants.

FCI values were computed for the right and left temporalis (TA) muscles (*n* = 48 each) and the right and left masseter (MM) muscles (*n* = 48 each). To avoid bias from side-specific results, data for TAs (*n* = 96) and MMs (*n* = 96) were combined for analysis. Data processing and analysis involved three stages: raw data management using Excel 2019 (Microsoft, Redmond, WA, USA), distribution analysis and significance testing with Statistica™ 13.3 (TIBCO Software Inc., Palo Alto, CA, USA), advanced analyses, including bootstrap methods, using R statistical software (version 4.1.1, R Core Team, Vienna, Austria) [37,38].

The data distribution was assessed using the Shapiro–Wilk test. Since distributions deviated from normal, the nonparametric Mann–Whitney U-test was used. The effect size was calculated using the formula $r=\frac{2(\bar{R_{1}\;}-\bar{R_{2})}}{n_{1+}n_{2}}$ [36,39].

Subsequently, interval analysis was conducted using the bootstrap method. Bootstrapping is a statistical technique that allows for estimating confidence intervals or standard errors without making assumptions about the data distribution [40]. This method was selected due to the nonparametric nature of the data. The bootstrap analysis was performed as follows: each bootstrap sample, denoted as $X^{\left(b\right)}$, was generated from the original data [40] through resampling with replacement, following the mathematical notation

$$X^{\left(b\right)}\;\sim \;X,\;i=1,2,3,\ldots ,\;n$$

For each bootstrap sample $X^{\left(b\right)}$, the mean $\left(X_{boot}^{\left(b\right)}\right)$ was computed as

$$X_{boot}^{\left(b\right)}=\frac{1}{n}\sum_{i=1}^{n}X_{i}^{\left(b\right)}$$

The distribution of the bootstrap means $\left(X_{boot}^{\left(b\right)}\right)$ was used to determine the confidence intervals. Due to the nonparametric nature of the data, a 95% confidence interval could not be applied [41]. Instead, the 2.5th and 97.5th percentiles were used to define the intervals according to the following notation [42]:

$$Lower\;bound=2.5({\bar{X}}_{boot})$$

$$Upper\;bound=97.5({\bar{X}}_{boot})$$

${\bar{X}}_{boot}$ represents values below the 2.5th percentile or above the 97.5th percentile. This allowed for the determination of values for the healthy group (values for TAs and MMs) and for the group with the painful muscular form of TMDs (values for TAs and MMs). Subsequently, the lower cutoff point was determined using the following formula:

$$Lower\;cut-off\;point=\frac{{Upper\;bound}_{TMDs\;}-{Lower\;bound}_{Healthy\;group\;}}{2}$$

The upper cutoff point was established based on the calculated upper bound, supplemented by a visual analysis of the data to identify the point of minimal overlap between truly positive and truly negative cases. This approach was adopted because masticatory muscle hyperactivity is also a symptom associated with dysfunction. For example, hyperactivity can occur in bruxism [4,43] and during stressful situations [44,45].

Sensitivity and specificity analyses for the obtained thresholds for the TAs and MMs were conducted using the following formulas [46]:

$$Sensitivity=\frac{True\;Positives}{True\;Positives+False\;Negatives}$$

$$Specificity=\frac{True\;Negatives}{True\;Negatives+False\;Positives}$$

## 3. Results

The data analysis revealed statistically significant differences with a large effect size. Healthy individuals had higher FCI values compared to those with TMDs. Additionally, TA FCI values were lower than MM FCI values, regardless of the group. The healthy group had an FCI TA score of 76.46, while the TMDs group scored 52.95. In comparing the MM FCI, the healthy group scored 83.63, whereas the TMDs group scored 61.60 (Table 1).

Following the methodological framework, thresholds were established for both groups. For the healthy group, the TA FCI range was determined to be between 60.48 and 82.62, while for the TMDs group, the TA FCI range was between 35.14 and 55.69. The MM FCI range for the healthy group was between 69.14 and 99.09, whereas for the TMDs group, it was between 48.09 and 74.47 (Table 2).

Healthy participants exhibited higher threshold values compared to individuals with TMDs. Sensitivity and specificity analyses showed that both thresholds had identical specificity values, higher than their corresponding sensitivity values (Table 3).

## 4. Discussion

This study represents the first attempt to determine ranges for electromyographic patterns within masticatory muscle activity. Additionally, TA FCI values were lower than MM FCI values, regardless of the group. The healthy group had a TA FCI score of 76.46, while the TMDs group scored 52.95. When comparing the MM FCIs, the healthy group scored 83.63, whereas the TMDs group scored 61.60. The observed statistical differences demonstrate the effectiveness of the FCIs in distinguishing the studied population [26]. Based on these differences, FCI ranges were determined: the lower bound was based on statistical analyses, while the upper bound was established according to the described methodology. The upper cutoff point was determined based on the calculated upper bound, supplemented by a visual analysis of the data to identify the point of minimal overlap between truly positive and truly negative cases, highlighting that bioelectrical hyperactivity is also not beneficial [43,44,45].

Based on the presented research, the FCI ranges for healthy, pain-free individuals for the TA were between 58 and 145, while for the MM, between 72 and 210. Lower values may indicate muscle activation disorders and occur in patients with the painful, muscular form of TMDs. The established thresholds demonstrated higher specificity levels, suggesting that these ranges could identify healthy individuals with 69% accuracy. Using these thresholds in clinical conditions makes it possible to monitor and predict the reaction of the muscular system in response to orofacial pain.

The Pain Adaptation Model demonstrates that changes in muscle activity limit movement and protect tissues from further injury [47]. Thus, in painful TMDs (e.g., myalgia, myofascial pain), the masticatory muscle has an incorrect activation pattern due to the pain condition, which disrupts both resting and clenching muscle activity. This condition may increase electromyographic values during the resting mandibular position and reduce sEMG potentials during teeth clenching [48]. A change in these parameters can be detected during an sEMG examination, and the results can be analyzed using the FCI. Using the FCI ranges for TMDs and healthy individuals, classifying study subjects or patients into a predisposition group based on the sEMG examination, may have significant clinical value. Hence, a prospective cohort study using electromyographic assessments may confirm the assumptions of specific ranges for the FCI. Moreover, the diagnostic thresholds proposed in this study will allow for exploring ranges for other patterns, such as the ACI, ASI, and POC [27,28].

As noted in the introduction, TMDs are also associated with economic challenges. The treatment of TMDs involves significant expenses for patients as well as for national budgets [17]. The treatment of TMDs creates an annual economic burden of approximately USD 4 billion worldwide [18].

To mitigate these substantial costs for both patients and governments, the development of additional diagnostic tools is crucial. These tools would help identify at-risk groups and facilitate effective prevention [49]. The defined ranges could serve as a screening tool, allowing for the identification of patients at risk before pain arises and dysfunction occurs. This would enable early referral for observation and further monitoring of TMJ function.

On the other hand, TMDs are characterized by a complex etiology [2], and pain-related factors may be influenced by variables such as hormonal fluctuations in women [10,11], adding to the diagnostic challenges. Additionally, defining an upper threshold for bioelectrical hyperactivity is complex as it may also relate to changes in the stomatognathic system or anatomical differences. Therefore, assessing and classifying a patient with TMDs must be approached individually, considering the interviews, thorough clinical diagnostics, imaging tests, assessment of comorbid disorders, and electromyographic examination.

At the end of this discussion, it is also important to consider the potential of combining diagnostic methods for TMJ. This article focuses on electromyographic testing and determining thresholds for electromyographic patterns. Surface electromyography is a quick, non-invasive, and cost-effective diagnostic method for masticatory muscles [19,20]. Another commonly used diagnostic method is ultrasound imaging [50,51,52]. Both methods can effectively complement each other, providing a complete clinical picture of the patient. The combination of these two methods can help determine whether, in addition to changes in bioelectric activity reflected by alterations in electromyographic patterns in patients with TMDs [1], structural changes in the TMJ, as visualized through ultrasound, will also be observed.

The primary limitation of this study is the lack of ROC curve (Receiver Operating Characteristic) [53] analysis and calculation of AUC (Area Under Curve) [54,55]. While the ROC and AUC are useful for diagnostic tests or classifiers with a single cutoff point, they are unsuitable for tests defining intervals rather than discrete thresholds. When tests define results as a range rather than a single point, assigning a clear decision threshold becomes challenging, rendering ROC curve construction impractical. The effectiveness of such tests depends on multiple parameters, such as the beginning and end of the range, which the AUC cannot adequately address.

## 5. Conclusions

The proposed FCI ranges, calculated from RMS sEMG amplitude values for healthy individuals, are as follows: TAs: 58 to 145; MMs: 72 to 210. Lower values may indicate muscle activation disorders and occur in patients with the painful, muscular form of TMDs. This study is the first to propose diagnostic thresholds for electromyographic indices, and we recommend interpreting these findings with caution while encouraging replication in larger, more diverse samples to account for cultural and demographic variations.

## Figures and Tables

**Table 1:** Comparison of FCI results between groups.

	Mean TA FCI	SD	Mean MM FCI	SD	Z	p	ES
Healthy Group	76.46	58.55	83.63	76.87	4.14	0.00	96.5
TMDs Group	52.95	55.87	55.11	61.60	3.74	0.00	96.5

TMDs—temporomandibular disorders; FCI—functional clenching index; TA—temporalis anterior; MM—superficial part of the masseter muscle; ES—effect size.

**Table 2:** Determination of intervals corresponding to a 95% confidence interval.

	Lower Bound TA FCI	Upper Bound TA FCI	Lower Bound MM FCI	Upper Bound MM FCI
Healthy Group	60.48	82.62	69.14	99.09
TMDs Group	35.14	55.69	48.09	74.47

TMDs—temporomandibular disorders; FCI—functional clenching index; TA—temporalis anterior; MM—superficial part of the masseter muscle.

**Table 3:** Presentation of adjusted FCI intervals.

	Lower Bound FCI	Upper Bound FCI	Sensitivity	Specificity
TA	58	145	61%	69%
MM	72	210	58%	69%

FCI—functional clenching index; TA—temporalis anterior; MM—superficial part of the masseter muscle.

## Data Availability

The original contributions presented in this study are included in the article. Further inquiries can be directed to the corresponding author.
